# N doping of TiO_2_ nanocrystal for efficient photodegradation of organic pollutants under ultraviolet and visible light irradiation

**DOI:** 10.3906/kim-2103-7

**Published:** 2021-05-30

**Authors:** YU Xiaoyou, LV Qianrui

**Affiliations:** 1 Beijing Energy Technology Co. Ltd., Beijing, China; 2 School of Science, Beijing Jiaotong University, Beijing, China

**Keywords:** photocatalysis, nanocrystals, nitrogen doping, titanium oxide, organic pollutants

## Abstract

To decrease the band gap of TiO_2_ and enhance its photocatalytic performance, in this work, N-doped nanocrystalline TiO_2_ is synthesized successfully through a simple sol-gel synthesis process with dicyandiamide used as N source. The N-doped TiO_2_ photocatalysts have been characterized in detail by means of powder X-ray diffraction, Raman spectroscopy, infrared spectroscopy, UV-vis spectroscopy, X-ray photoelectron spectroscopy, and scanning electron microscope. The photocatalytic activities of N-doped TiO_2_ to different organic pollutants are significantly different. Using 2 × 10^−5^ mol/L methylene blue as the target pollutant, N-doped TiO_2_ can produced approximately 100% conversion within 40 min, the photocatalytic degradation ability of which is equal or superior to commercial or bare TiO_2_. The ameliorated photocatalytic activity of N-doped TiO_2_ photocatalysts is attributed to the appropriate crystallinity, good dispersibility, modified Zeta potential, and increased absorption.

## 1. Introduction

Photocatalytic technology is a robust method to solve environmental pollution because of its green, safety and high efficiency. As a promising photocatalyst, TiO_2_ possesses admirable merits such as low cost, high corrosion resistance, and nonhazardous nature. As a result, it has been widely used in the fields of pollutant treatment, hydrogen generation via photocatalytic water splitting and self-cleaning [1–5]. However, the high band gap energy (3.2 eV) of TiO_2_ corresponds to the wavelength of ultraviolet light (<387 nm), [6] which significantly hinders the full use of solar light because it can only capture approximately 5% of total solar irradiation [6].

Atom doping is regarded as a common approach to decrease the TiO_2_ band gap [7–15]. Especially, substituting some of the oxygen atoms by nitrogen atoms is an effective method because the resulting mixed states of substitutional N 2p and O 2p can narrow the band gap of TiO_2_ so that electrons can be excited from the valence band to the conduction band by the irradiation of visible light [7,16]. In addition, oxygen vacancies produced by N-doping can effectively boost the photocatalytic performance of TiO_2_ because oxygen vacancies can induce the rearrangement of the surrounding atoms, affecting the concentration of the surface hydroxyl and ameliorating the detachment and transfer of photo-generated carriers [17–23]. Previous studies have shown that the performance of N-doped TiO_2_ is closely related to the raw materials and synthesis methods. For example, Asahi et al. claimed that nitrogen doping was helpful for the photoactivity of TiO_2_ under visible light, without weakening the photoactivity under ultraviolet light [7]. However, N-doped TiO_2_ prepared by other methods showed the degradation of photoactivity under ultraviolet irradiation [24–26]. Therefore, people have been exploring various doping methods for several years. At present, the common N doping methods include hydrothermal method [27–28], solvothermal method [29], high temperature calcination [30], sol-gel method [31], and so on. Among them, the hydrothermal method can make a large amount of N doping into TiO_2_ nanoparticles; the solvothermal method can achieve the synthesis of nanoparticles with higher grain size and narrower particle size distribution; The calcination method is helpful to change the physical and chemical properties of the crystal, making it easier for the N to enter the TiO_2_ crystal lattice. Recently, Ma et al. reported the N-doped TiO_2_ via the vapor-thermal method, using urea as the N source [32]. It showed great enhancement in the adsorption coefficient and photocatalytic degradation ability.

Searching for novel synthesis methods of high-performance N-doped TiO_2_ photocatalysts is still of great interest. In this work, we use dicyandiamide rather than common urea or ammonia as N source to prepare N-doped TiO_2_ nanoparticles by sol-gel method. The method is relatively simple and a series of characterizations are used to prove the successful existence of N in TiO_2_ lattice. The as-prepared N doped TiO_2_ are used for the photocatalytic degradation of a series of organic pollutants under ultraviolet and visible light irradiation. It is found that the N doping not only improves photocatalytic activity for the photodegradation under ultraviolet light but also expands the absorption of TiO_2_ in visible light and show good visible light photocatalytic activity. The N-doped TiO_2_ shows equal or superior activity to commercial P25 or bare TiO_2_ for the decontamination of methylene blue (MB). The effect of nitrogen doping amounts on the photodegradation ability and the difference in the degradation of different organic pollutants are also discussed.

## 2. Materials and methods

All reagents used in this research were analytically pure and used without further purification.

Glacial acetic acid was added to a beaker containing anhydrous ethanol at room temperature. Then the stoichiometric n-butyl titanate was add and stir for 20 min to make it evenly mixed to obtain a light yellow solution. Then stoichiometric dicyandiamide was dissolved in 150 mL of deionized water and mix with the above light yellow solution at 30 °C and stirred for 24 h. After aging for 2 days, freeze-dry was taken. The product was putted into an oven at 80 °C for 1 h, and then placed in a muffle furnace at 530 °C for 2 h.

Dicyandiamide as N source were added to the Ti gel. After calcination, TiO_2_ nanoparticles with different nitrogen doping amounts were prepared, which were denoted as NT-1, NT-2, NT-3, and NT-4. The raw mole ratios of n-butyl titanate to dicyandiamide for NT-1, NT-2, NT-3 and NT-4 are 1.2, 0.6, 0.3, and 0.15 respectively.

SEM analysis was carried out using a field-emission scanning electron microscope Hitachi SU8010. TEM analysis was carried out by JEOL-2100F. The phase structure was detected by X-ray diffractometer Rigaku D/MAX-RB X-ray diffractometer. The diffractograms were recorded in a 2θ range between 10° and 80° in steps of 0.02 °. Raman spectroscopy was collected using the confocal Raman spectrometer Renishaw RM2000. The laser wavelength was 514 nm and the laser optical power was 0.5 mW. The IR spectrum was collected using the Perkin Elmer System 2000 infrared spectrometer. UV-vis spectrum in the region 250–800 nm was collected using Hitachi U-3010 ultraviolet-visible spectrophotometer.

The photocatalytic performance of the catalyst was evaluated by the liquid phase degradation of organic pollutants MB, rhodamine B (RhB), methylene orange (MO), phenol and dichlorophenol under ultraviolet light. The UV reaction used a 100 W (λ = 365 nm) mercury lamp. 25 mg of NT nanoparticle powder was put into 50 mL of organic pollutant (C_0_ = 2 × 10^−5^ mol/L) solution. Then stirring for 1h made the probe molecules reach adsorption equilibrium on the surface of NT particles. When the light was turned on, the photocatalytic degradation reaction was carried. Samples of 2.5 mL were taken at different irradiation times and they were centrifuged (4000 rpm, 10 min) to separate NT particles. Then the supernatant was taken to detect the absorbance of colored organic pollutants (MB, RhB, MO) with a UV-vis spectrophotometer. High performance liquid chromatography was used to detect the electrical signals of DCP and phenol, and then the concentration changes of the target molecules in the solution were tracked.

The visible light catalytic activity of catalysts was evaluated by degrading MB molecules in water. A 500 W xenon lamp was used as the light source, and a filter (λ > 420 nm) was used to filter out ultraviolet light below 420 nm. Fifty mL of 3 × 10^−5^ mol/L MB solution was put into a quartz tube, and 25 mg of N-doped TiO_2_ nanoparticles was added. Stirring for 20 min made MB molecules reach an adsorption equilibrium on the surface of N-doped TiO_2_ particles. When the light was turned on, the photocatalytic degradation reaction was carried. Three mL of sample was taken at different irradiation times and centrifuged to separate the N-doped TiO_2_ particles (4000 rpm, 10 min). The supernatant was taken and the absorbance was detected by a UV-vis spectrophotometer.

A 500 W xenon lamp was used as the light source, and a filter (λ > 420 nm) was used to filter out ultraviolet light below 420 nm. The electrochemical workstation CHI660B (Shanghai Chenhua Instrument Company) was used for the photoelectric performance test of the three-electrode system, which was achieved in a self-made three-electrode electrolytic cell with quartz window. The counter electrode is Pt wire; the reference electrode was a saturated calomel electrode; the working electrode was an N doped-TiO_2_ membrane electrode; and the electrolyte was a 0.1 mol/L Na_2_SO_4_ solution. The scanning speed of the linear scanning measurement photocurrent is 50 mV/s.

## 3 Results and discussion

### 3.1. Characterization of catalysts

**Figure 1:**
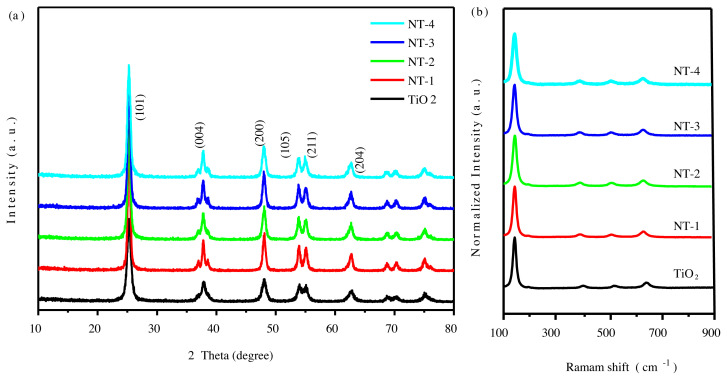
XRD patterns (a) and Raman spectra (b) of the anatase N-doped TiO_2_ nanoparticles.

The crystal structures of TiO_2_ with different nitrogen doping amounts prepared by sol-gel method were characterized by X-ray diffraction (XRD). As shown in Figure 1a, XRD spectra of all samples have similar spectral characteristics. It indicates that all samples crystallize as the single-phase anatase structure (JCPDS 21-1272) and the crystal shape of TiO_2_ did not change after nitrogen doping. The peaks of N-doped TiO_2_ show slightly shift to a smaller angle. It is speculated that the doping nitrogen atom with large atomic radius replaces the oxygen atom in TiO_2_, which makes the interplanar spacing in TiO_2_ become larger. Raman spectroscopy is a sensitive technique to distinguish individual phase of TiO_2_. Raman spectroscopy was exploited to further explore crystal properties of N-doped TiO_2_. Figure 1b demonstrates the well-defined peaks located at 143 cm^−1^, 396 cm^−1^, 516 cm^−1^, and 638 cm^−1^ can be respectively assigned the vibrational modes of E_g_, B_1g_, B_1g_+ A_1g_ and E_g_ of the anatase phase. No other obvious new peaks appear in the Raman spectrum after doping N, indicating the nanoparticles contain exclusively anatase [33].

**Figure 2:**
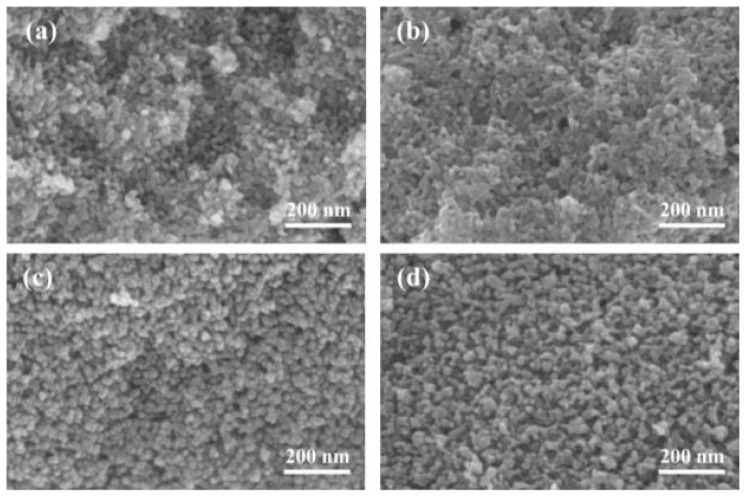
SEM image of the anatase N-doped TiO_2_ nanoparticles of NT-1(a), NT-2(b), NT-3(c) and NT-4(d).

**Figure 3:**
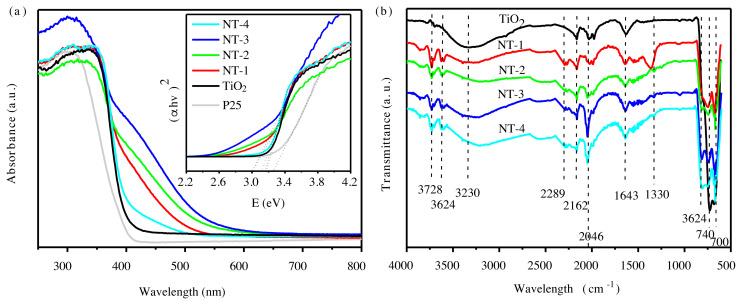
UV-vis absorbtion spectra (a) and infrared spectra (b) of N-doped TiO_2_.

Revealed in the SEM images (Figure 2) and TEM image (Figure S1), the N-doped TiO_2_ particles look spherical, with similar nanoparticle sizes of about 15 nm and good dispersity. Figure 3a shows the UV-visible absorption spectrum of the samples. It can be seen that the absorption of N-doped TiO_2_ is generally stronger than that of TiO_2_. Particularly, the N doping causes stronger absorption capacity in the visible light region, and we can see the red shift of the absorption edge, which indicates that the band gap of TiO_2_ is narrowed. The bandgaps calculated from the Tauc plots of bare TiO_2_, NT-1, NT-2, NT-3, NT-4 are 3.22eV, 3.19 eV, 3.13 eV, 3.06 eV, 3.20 eV. It can be deduced that the 2p states of the doped nitrogen atoms mixing with O 2p states contributed to the band gap narrowing. Due to the narrower band gap, an electron could be excited easily from the valence band to the conduction band in the N-doped TiO_2_, which is helpful in increasing the photocatalytic activity. In other side, the absorption (bandgap) increases (decreases) as N-doping increases from NT-1 to NT-3. However, in the NT-4, the absorption (bandgap) decreases (increases) again, which suggests that proper amount of doping is important. The X-ray photoelectron spectroscopy (XPS) was carried out to scan the binding energy range of N 1s (Figure S2). It exhibits the binding energy of N 1s at 397 eV, originating from Ti-N bonds in TiO_2_ lattice [23,34], whereas the peak at 400 eV was consistent with the adsorbed nitrogen N_2_ on the surface of TiO_2_ [23].

Figure 3b shows the infrared spectra of the samples. The wide absorption peak at 3230 cm^−1^ is caused by the stretching vibration of O-H. The absorption peak at 1643 cm^−1^ is caused by the bending vibration of O-H. The above two peaks exist both in TiO_2_ and N-doped TiO_2_, and the peak in N-doped TiO_2_ is strengthened. It needs to be noted that there are several new peaks in N-doped TiO_2_, located at 3624, 3728 cm^−1^, 2289 cm^−1^, 1330 cm^−1^ and 700 cm^−1^. The peaks at 3624, 3728 cm^−1^ and 2289 cm^−1^ correspond to the N-containing groups adsorbed on the surface of TiO_2_ nanoparticles, i.e. –NH_2_ and –CN in dicyandiamide. The peak at 1330cm^−1^ is presumed to be the characteristic absorption peak of Ti-OH. The characteristic absorption peak at 700 cm^−1^ is derived from the N-Ti-O structure formed by N atoms replacing O atoms in the TiO_2_, which indicates that N atoms not only exist in the gaps of TiO_2_ lattice, but also replace part of the O atoms in the TiO_2_ lattice.

### 3.2. Photocatalytic activity

**Figure 4:**
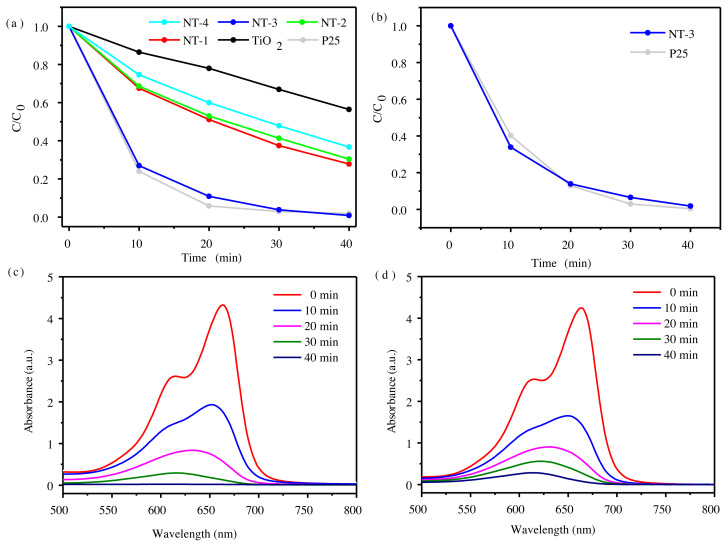
(a) Photocatalytic degradation of 2 × 10^−5^ mol/L MB aqueous solution by N-doped TiO_2_ and P25. (b) Photocatalytic degradation of 3 × 10^−5^ mol/L MB aqueous solution by NT-3 and P25. UV-vis absorption spectrum of MB aqueous solution with N-doped TiO_2_ (c) and P25 (d) photocatalysts at different degradation time; the initial concentration of MB is 3 × 10^−5^ mol/L.

The photocatalytic activity of the samples was measured in terms of the relative concentration of target pollutants. The colored target pollutants have typical absorbance peaks, and consequently their concentrations can be readily determined by a UV–vis spectrophotometer. Firstly, with 2 × 10^−5^ mol/L MB as the target pollutant, the degradation ability of the N-doped TiO_2_ catalyst under ultraviolet light was tested, as shown in Figure 4. It is found that all the N-doped TiO_2_ photocatalysts display superior photocatalytic behaviors to the bare TiO_2_. The activity of N-doped TiO_2_ gradually increases as the doping concentration going up, then tends to drops, which suggests that excessive N will depress the activity. NT-3 present the far greater degradation activity than that of other samples of the same series prepared by the sol-gel method, and even comparable to the performance of P25 prepared by the flame method from the commercial company. It produced approximately 100% conversion of MB within 40 min (Figure 4a). In order to further study the ultraviolet activity of NT-3 and P25, we then increased the MB concentration to 3 × 10^−5^ mol/L. Figure 4b shows the evolution of MB concentration at each time interval, which are derived from the absorption spectra (Figures 4c & 4d). It can be seen that NT-3 and P25 degrade MB simultaneously. The time it takes for NT-3 to completely degrade MB is almost the same as that of P25.

**Figure 5:**
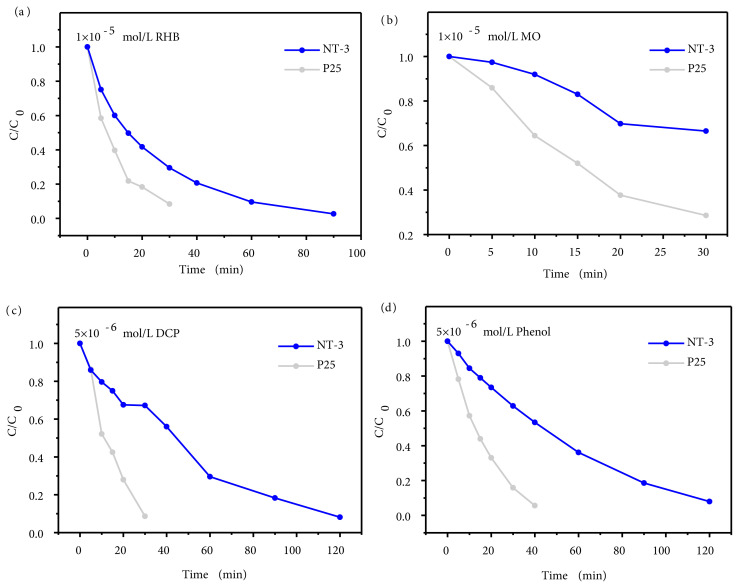
Photocatalytic degradation of RHB (1 × 10^−5^ mol/L) (a), MO (1 × 10^−5^ mol/L) (b), DCP (5 × 10^−6^ mol/L) (c), phenol (5 × 10^−6^ mol/L) (d) aqueous solution by N-doped TiO_2_ and P25.

The ultraviolet activity of N-doped TiO_2_ to other target molecules are also tested and show different results. For organic color dyes, Figure 5a shows the degradation of RhB under UV light. NT-3 takes 3 times as much time to degrade RhB completely as P25. Figure 5b shows the degradation of MO under UV light. After 30 min of light reaction, neither of NT-3 and P25 can completely degrade it, and the degradation ability of NT-3 is also worse than that of P25. MB and RhB are cationic dyes, while MO is an anionic dye. The results show that NT-3 is more capable of degrading cationic dyes than anionic dyes. In order to explain the selectivity of degrading organic color dyes, a series of samples were tested for Zeta potential, as shown in Table. The NT-3 is the most negative, indicating that it has a strong negative charge and is easy to attract cations. It is speculated that after the cationic dyes on the surface of NT-3 are degraded under ultraviolet light, the surrounding dye ions can be quickly supplemented to the catalyst surface because of the electrostatic attraction, thus accelerating the degradation rate and show the selectivity.

For organic colorless pollutants, almost complete degradation is achievable. While, NT-3 takes 4 times as much time to degrade dichlorophen (DCP) completely as P25 (Figure 5c) and takes 3 times as much time to degrade phenol completely as P25 (Figure 5d). As can be seen, the degradation ability of NT-3 is reduced for these colorless small-molecular organics compared with the degradation of MB.

**Figure 6:**
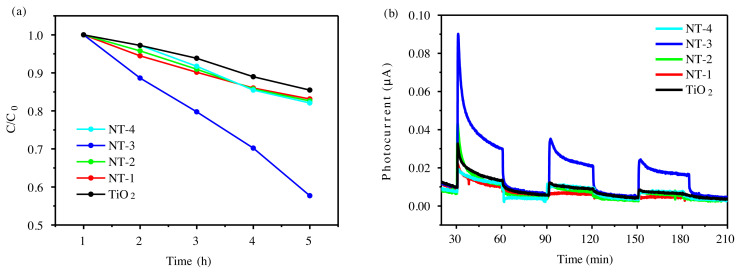
(a) Photocatalytic degradation of 3 × 10^−5^ mol/L MB aqueous solution by N-doped TiO_2_ under visible light. (b) Photocurrent of N-doped TiO_2_.

Taking MB as the target pollutant, the degradation ability of the catalysts under visible light was also tested, as shown in Figure 6a. NT-3 has the strongest degradation activity, which is consistent with the strongest degradation activity under ultraviolet light, indicating that the negatively charged nature of the material is also conducive to the adsorption-degradation under visible light. The good photocatalytic activity of NT-3 is related to the efficient absorption of light and the separation of photogenerated electrons and holes. We further analyze it through the photocurrent test. Figure 6b shows the photocurrent of N-doped TiO_2_ photocatalysts under visible light. It indicates that the photoresponse current of the NT-3 sample is the strongest, which is consistent with the best photocatalytic activity of NT-3 in the N-doped TiO_2_.

Phenol is a typical organic pollutant with high resistance to direct photolysis and oxidation under visible light. Therefore, we also use phenol as a degradation object under visible light. As shown in Figure S3, phenol can be degraded to a certain extent by N-doped TiO_2_, bare TiO_2_ or P25. Although the appropriate amount of N doping can make the photocatalytic performance much better than bare TiO_2_ or P25, N-doped TiO_2_ can only degrades ~10% of the phenol in seven hours. Compared with the above organic pollutants, the degradation efficiency of phenol is low. To achieve efficient degradation of phenol under visible light, it may be necessary to further modify the photocatalyst or combine with other catalytic methods.

## 4. Conclusion

N-doped TiO_2_ was favorably synthesized by a simple sol-gel method, with dicyandiamide used as N source. Doping N into TiO_2_ boosts the light response capacity and promotes the generation of carriers, consequently ameliorating the catalytic activity, especially visible light responsive photocatalytic activity. The doping ratio possesses a considerable effect on the activity of TiO_2_. The resulting TiO_2_ doped with an appropriate amount of N exhibits high photocatalytic activity for the degradation of MB under ultraviolet and visible light, which is attributed to the appropriate dispersibility and crystallinity, modified zeta potential and enhanced absorption. This work provides a feasible route for improving the visible photocatalytic activity of nonmetal-ion-doped TiO_2_, which show the potential application to other oxides with wide bandgap energies.

## Supplementary Data

Figure S1 TEM image of NT-3

Figure S2 XPS spectra of N-doped TiO_2_

Figure S3 Photocatalytic degradation of 5 × 10^−6^ mol/L phenol aqueous solution by N-doped TiO_2_ under visible light.

## Figures and Tables

**Table:** Zeta potential of N-doped TiO_2_ and P25.

Sample	NT-1	NT-2	NT-3	NT-4	TiO_2_	P25
Zeta potential (mV)	−64.7	−53.8	−71.7	−55.1	−9.2	−10.1
