# Supplementary figures and images for: N doping of TiO2 nanocrystal for efficient photodegradation of organic pollutants under ultraviolet and visible light irradiation
Source: Turk J Chem. 2021 May 30;45(5):1366–74. doi: 10.3906/kim-2103-7 (PMC13061293; doi:10.3906/kim-2103-7)

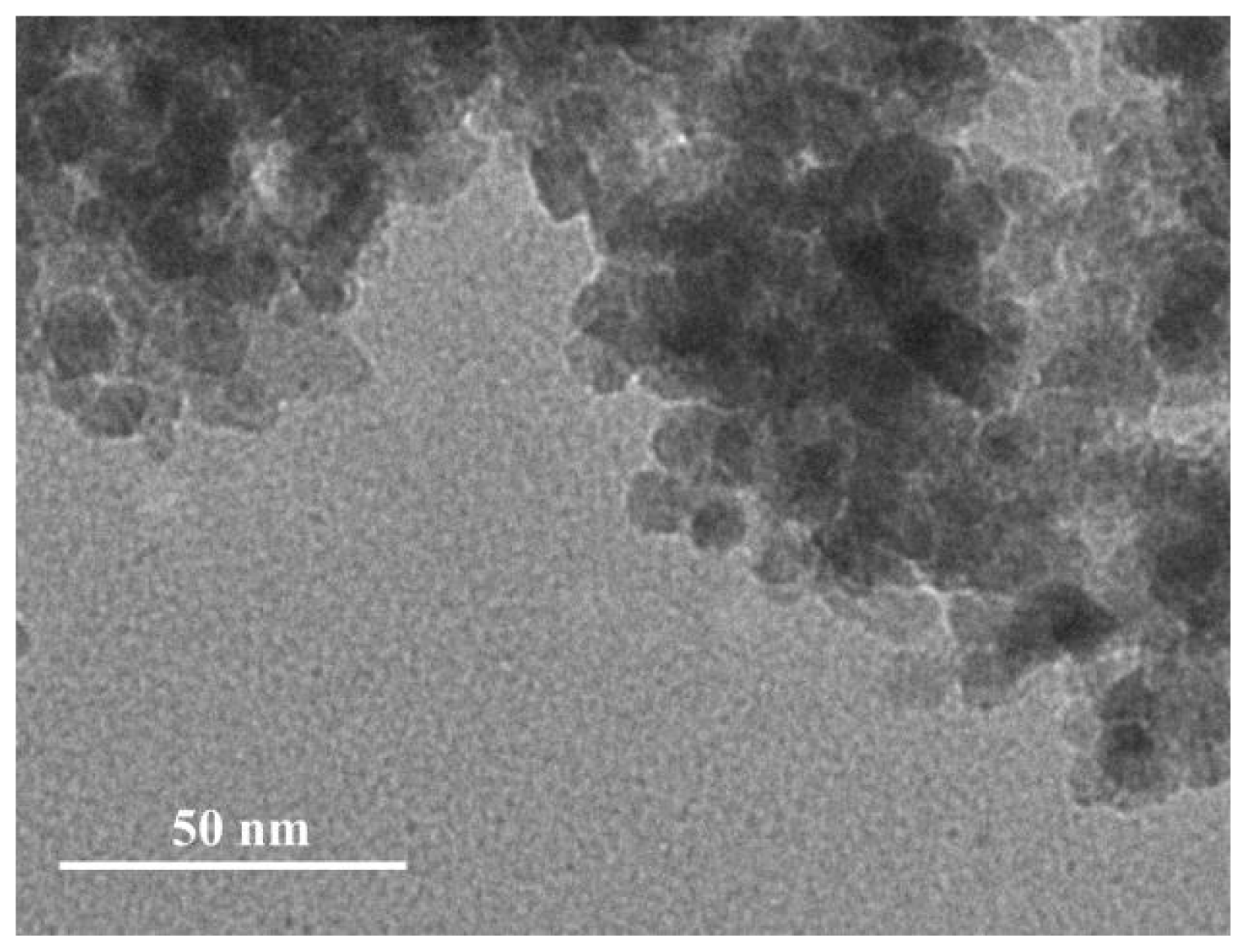

Supplement: Figure S1 — TEM image of NT-3 [file tjc-45-05-1366s1.tif]

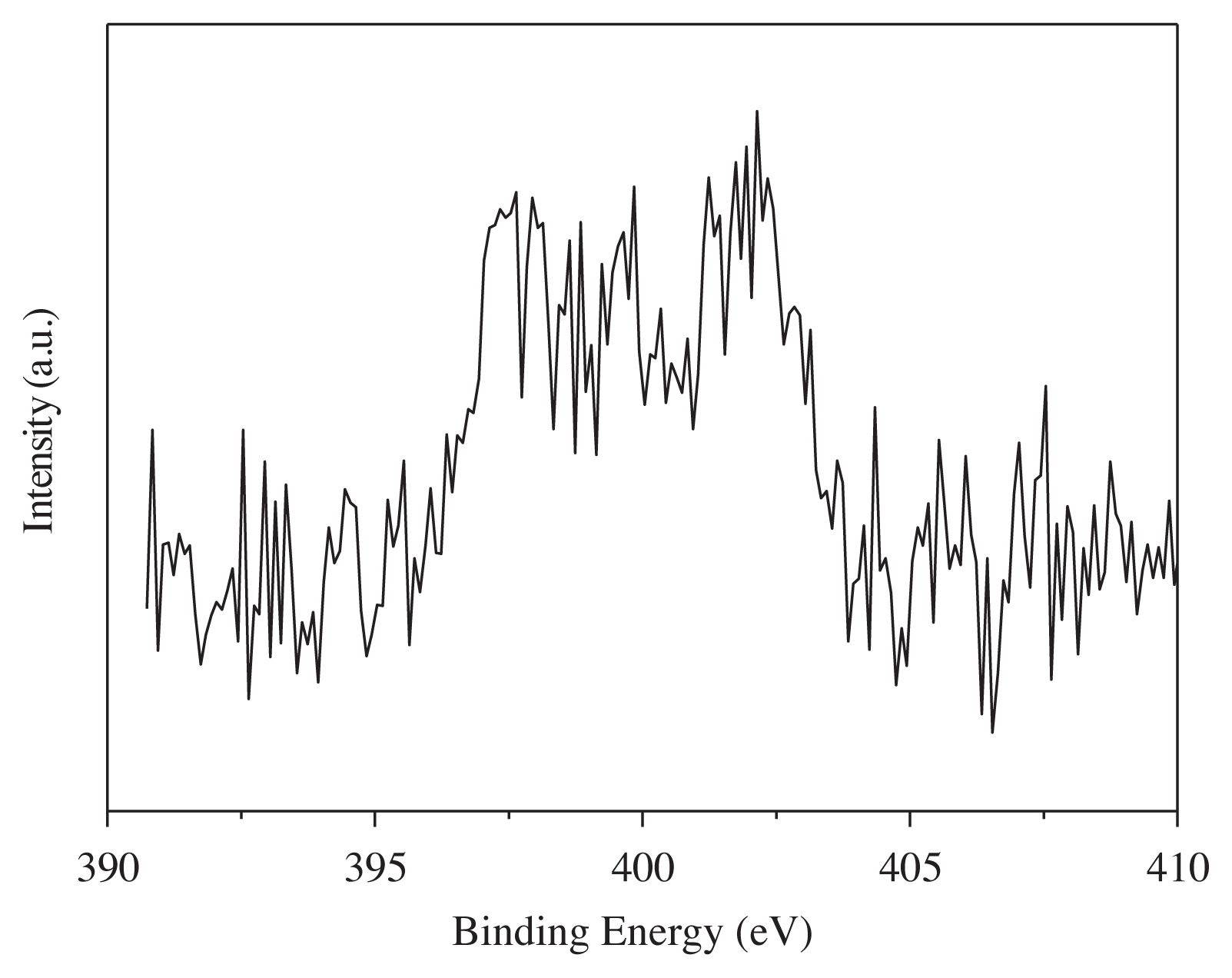

Supplement: Figure S2 — XPS spectra of N-doped TiO2 [file tjc-45-05-1366s2.tif]

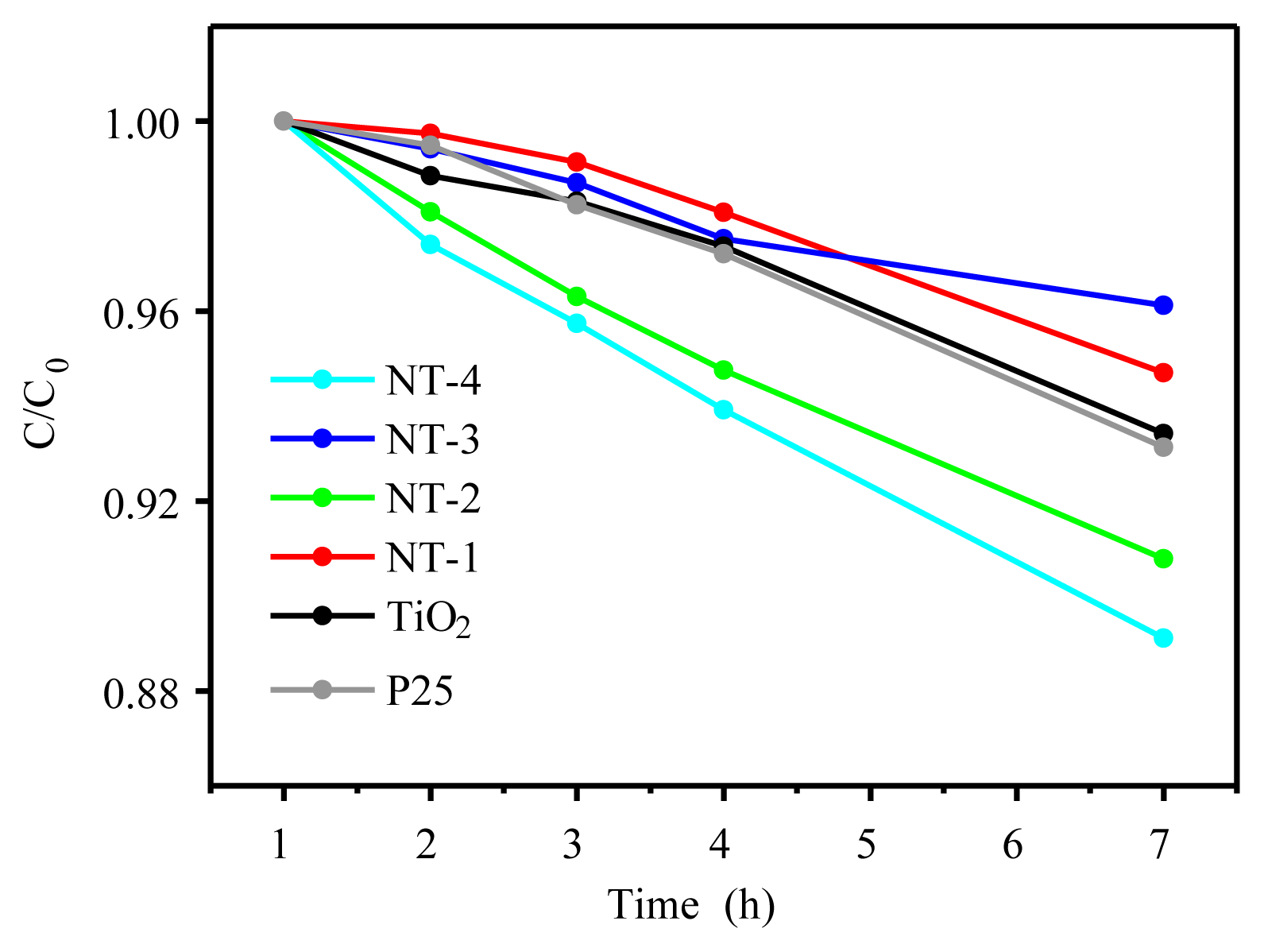

Supplement: Figure S3 — Photocatalytic degradation of 5 × 10^−6^ mol/L phenol aqueous solution by N-doped TiO2 under visible light. [file tjc-45-05-1366s3.tif]
